# More Room, or Better Room?

**Published:** 1892-04-09

**Authors:** 


### The Hospital

An Institutional Journal of

**Science, Medicine, Nursing, and Philanthropy.**

*For Hospitals, Asylums, Infirmaries, Dispensaries, Medical Practitioners, Students, Nurses, and the Charitable Public.*

Vol. XII.—No. 289.] [April 9, 1892.

### More Room, or Better Room?

Is it the function of biological science, or indeed of any science, merely to accumulate knowledge? If a historical answer may be given to this question, one would say decidedly not. To take the men of our own time, we find those who are most eminent in science most anxious to make their knowledge of practical value to mankind; and we perceive that their eminence is generally proportionate to the energy and persistency of the efforts they have made to illuminate the minds of all men, and to bring all men to what they have considered the right way of thinking. As examples of this may be recollected Professor Huxley and Professor Tyndall in this country, Mr. Edison in America, and M. Pasteur in Paris, with many others whose names will be readily thought of. "Knowledge for the sake of knowledge" has a pleasing sound to some ears. But let us convert the same idea into other terms, and then consider it. For example, "money for the sake of money," "roast beef for the sake of roast beef," "trousers for the sake of trousers." One can manage to get up a little sympathy with the miser who accumulates money because he loves it; but with the man who should fill his larder and his kitchen and his reception-rooms as well with joints of unutilised roast beef, or his wardrobes and all his upper rooms with thousands of pairs of trousers for the sake of trousers, one feels that there is one thing, and one thing only, to be done: he must be promptly provided with a strait waistcoat. To the generous mind "knowledge for the sake of knowledge," if accepted literally, is just as ridiculous as "trousers for the sake of trousers."

It is a part of the business of the biologist and of the physiologist to consider all the subjects of their investigations in the mass as well as in the individual. To be truly scientific they must not think of a dog, but of dogs, of every family and class of dogs; and not only of dogs, but of dogs in their environment, dogs in their history, dogs in their present and their past, and dogs in their future. This is what the scientific breeder of horses and cattle, sheep and pigs, does. What marvellous results he produces! He effects more in the improving of the breed of animals in a quarter of a century than nature, unassisted, has effected in four or five centuries, or perhaps in four times four or five. Is it possible that the biologist and the physiologist do not recognise the far greater dignity of the study of man than of the study of the lower animals, and the far greater worth of valuable modifications in man, than of similar modifications in horses and sheep? Of course, for a man really to see and practically to purpose the improvement of the race of men on any considerable scale, would be to show an uncommon breadth, strength, and capacity of mind; and that is not to be expected every day, even in the physiological laboratory. But there have been known cattle breeders who, as the result of continuous study in the narrow laboratory occupied by their own limited herds, have been fired with a worthy ambition to improve the breed of stock throughout their own country and the world. One surely ought not to expect more comprehensiveness and greatness of mind in a mere cattle breeder, than in a scientific student of human physiology and animal life in general.

There are not wanting indications in various parts of the world that something of this kind is being looked for at the hands of workers in biological laboratories. The other day Mr. Thomas W. Palmer, President of the World's Columbian Commission, in addressing an assembly of gentlemen at dinner in connection with the projected Chicago Exhibition, showed how clearly he appreciated the pressure of the environment in which the English-speaking races now find themselves, and the practical necessity for some large and scientific measure of readjustment of existing conditions. Said Mr. Palmer: "The progenitors of the race which has dominated our civilisation started out fifteen hundred years ago from the shores of the Baltic as pirates. Unlike the other branches of the Teutonic strain, they did not adopt the civilisation of the people whom they conquered. They destroyed, they extirpated those whom they dispossessed. It took just two hundred years to do it. Their civilisation was not one of adoption; it was one of development and expoliation. They lived, they increased, they struggled for one thousand years, and then started out on another career, or conquest, or absorption on this continent. It took them a little over two hundred years to accomplish that; but now the race has reached a barrier. The law of its nature, which has hitherto underlain all other laws, the tireless quest for homes towards the setting sun, has now given way to the inevitable. The English-speaking people stand upon our western coast, and, with hand uplifted and shaded eyes, look in vain across a boundless sea for a pathway for the soles of their feet. The period of compression has begun. The character of our civilisation is changing. The unsatisfied demand for more room must be satisfied by the creation of better room. Our people must seek adjustment under changed conditions. It is of much consequence to us all how we shall adapt ourselves thereto. Shall we rise to a higher, or sink to a lower plane? Shall we look to force or to reason?"

The demand that is here made upon reason can only be properly made upon the reason of those who study man as a reasoning animal, in his actual nature, his environment, and his history; can only, in fact, be properly made in the first instance upon the physiologist and the biologist, the natural human providences of physical and mental life. The physiologist must see, he does see, that men and women can have no upward development when they are crowded together in poisonous atmospheres, and deprived of the satisfaction of their natural desires and wants in a thousand particulars. Not progressive development, but degeneracy and final worthlessness and extinction are the goals to which men are bound under such circumstances. It is plain that one of two things must be provided—a new environment or a better environment: in Mr. Palmer's words, "more room or better room." The physiologist may tell us that he has not made it his business to study man's environment, but only man himself. But he ought to have made it his business, and he must now make it his business, and that for the best of all reasons, that it is naturally and properly his business. Or if it be not, whose business is it?

Men and women cannot live and improve in such narrow and privatory conditions as the great English and American towns impose upon them. Is it for the statesman to provide them with wider or better environment? Undoubtedly it is. But the statesman must be instructed; he must have it forced upon his intelligence that not development and happiness, but degeneracy and explosiveness are the inevitable fruits of unwholesome and impossible conditions. It is the physiologist, and the physiologist alone, who can force these things upon the statesman's attention with luminous and convincing clearness. "More room, or better room," is the demand. But more room is practically impossible. We are, therefore, shut up to the alternative of better room. Is better room also impossible? By no means. The world can support at least twice its present population without difficulty if it will utilise its available resources to the best advantage.

In England we have a population of about twenty-five millions, and there is under cultivation about twenty-five million acres of land, excluding wastes, open spaces, grouse moors, and afforested lands. Each acre of fairly good soil, if made to produce its utmost by scientific methods, would support two persons. Even in England, therefore, which is almost ten times more densely populated than the remainder of the earth, we could support double our present population by high scientific methods of agriculture; and if those methods were adopted, and large numbers of our present town populations were withdrawn from factories and workshops to carry them out, the common people, in twenty-five years, would be twice as healthy, twice as prosperous, and ten times more contented and happy than they are at the present time. "The era of compression," says Mr. Palmer, "has commenced; and the compression is excessive, intolerable, and dangerous." This is obvious to the man who thinks even a little: it is obvious most of all to him who studies human nature at the side of the sick bed and in the hospital ward. The physiologist cannot take the place of the statesman; but he can and must so worry the statesman by his persistent reiteration of these dangerous facts as to make political life a burden until an adequate remedy is sought and found. Of doctrinaire physiologists and doctrinaire statesmen the world has had more than enough. What the present emergency demands is men of commanding intelligence, high motive, and lifelong purpose who will carry through to practical issue the work of making the world a fairly happy place to live in for men of industry and common intelligence.

